# Achieving deep intratumoral penetration and multimodal combined therapy for tumor through algal photosynthesis

**DOI:** 10.1186/s12951-024-02476-7

**Published:** 2024-05-06

**Authors:** Xuwu Zhang, Xinyue Zhang, Shiqi Liu, Weidong Zhang, Liang Dai, Xifa Lan, Desong Wang, Wenkang Tu, Yuchu He, Dawei Gao

**Affiliations:** 1 Nano-Biotechnology Key Lab of Hebei Province, Yanshan University, No.438 Hebei Street, Qinhuangdao, 066004 People’s Republic of China; 2 Applying Chemistry Key Lab of Hebei Province, Yanshan University, Qinhuangdao, 066004 People’s Republic of China; 3 Department of Pharmacy, The First Hospital of Qinhuangdao, Qinhuangdao, 066004 People’s Republic of China

**Keywords:** *Chlorella pyrenoidosa*, Tumor interstitial fluid pressure, Photosynthesis and water decomposition, Deep drug penetration

## Abstract

**Background:**

Elevated interstitial fluid pressure within tumors, resulting from impaired lymphatic drainage, constitutes a critical barrier to effective drug penetration and therapeutic outcomes.

**Results:**

In this study, based on the photosynthetic characteristics of algae, an active drug carrier (CP@ICG) derived from *Chlorella pyrenoidos*a (CP) was designed and constructed. Leveraging the hypoxia tropism and phototropism exhibited by CP, we achieved targeted transport of the carrier to tumor sites. Additionally, dual near-infrared (NIR) irradiation at the tumor site facilitated photosynthesis in CP, enabling the breakdown of excessive intratumoral interstitial fluid by generating oxygen from water decomposition. This process effectively reduced the interstitial pressure, thereby promoting enhanced perfusion of blood into the tumor, significantly improving deep-seated penetration of chemotherapeutic agents, and alleviating tumor hypoxia.

**Conclusions:**

CP@ICG demonstrated a combined effect of photothermal/photodynamic/starvation therapy, exhibiting excellent in vitro/in vivo anti-tumor efficacy and favorable biocompatibility. This work provides a scientific foundation for the application of microbial-enhanced intratumoral drug delivery and tumor therapy.

**Graphical Abstract:**

**Figure:**
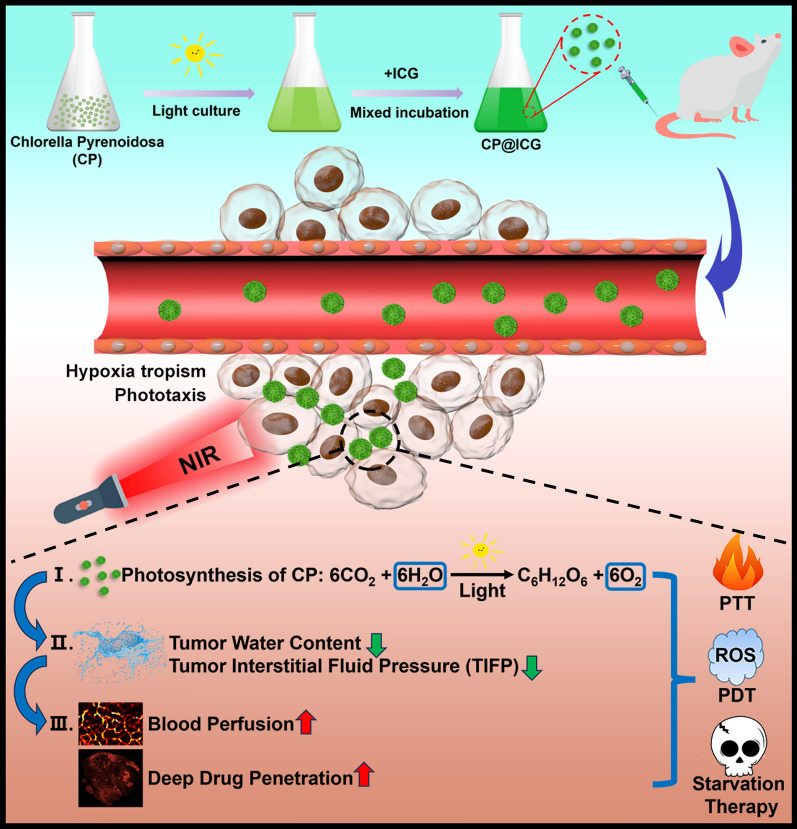

**Supplementary Information:**

The online version contains supplementary material available at 10.1186/s12951-024-02476-7.

## Background

In the course of cancer treatment, the prerequisite for achieving effective therapy lies in the efficient targeted delivery of drugs and their deep intratumoral penetration. However, early studies have indicated that only 0.7% of the injected dose of nanomedicines reaches the tumor site [1]. To address this issue, researchers have employed various strategies. These include surface modification of carriers with tumor cell-specific receptors to achieve targeted drug delivery to the tumor [2, 3]. Alternatively, structural alterations of the carrier can be implemented to transition from larger to smaller morphologies, thereby enhancing intratumoral penetration efficiency [4, 5]. Additionally, utilizing endocytosis facilitates carrier transmission between tumor cells, enabling deep-seated delivery of the carrier to the tumor [6, 7]. In our earlier investigations, our team observed that apart from the inherent properties of the carrier, the significantly elevated interstitial fluid pressure at the tumor site greatly hinders the efficiency of drug delivery. Lowering the interstitial pressure can substantially enhance the penetration of drugs within the tumor [8–10].

The fundamental reason for the low accumulation of drugs within the tumor is the insufficient blood perfusion within the tumor interior [11]. Studies have revealed that the local capillary venous pressure within tumors is approximately 20 mm Hg, while the interstitial pressure ranges from 20 to 130 mm Hg [12]. This discrepancy arises from the malignant proliferation of tumor cells and the lack of intratumoral lymphatic vessels, resulting in inadequate lymphatic drainage. This leads to an excessive accumulation of interstitial fluid within the cellular interstices, culminating in markedly elevated intratumoral interstitial fluid pressure [13, 14]. This counter-pressure differential severely impedes the perfusion of blood into the tumor interior. The primary component of tumor interstitial fluid is water, and its content correlates positively with interstitial fluid pressure. When the local interstitial fluid pressure within the tumor is at 15 mm Hg, its water content can reach up to 88% [15]. Consequently, in our team's earlier research efforts, we designed a series of nano-drug delivery systems based on catalytic materials for water decomposition. By utilizing processes such as photocatalysis and piezoelectric catalysis to decompose excess water within the tumor, we were able to subsequently reduce interstitial fluid pressure. This led to an improvement in local blood perfusion within the tumor, enhancing the delivery efficiency of nano-drugs within the tumor [8, 9, 16]. These findings provide ample evidence for the feasibility of this therapeutic strategy.

However, the aforementioned treatment process necessitates repeated intravenous drug administration, and its long-term adverse effects and potential risks within the body warrant further investigation. In contrast, selecting naturally occurring microorganisms with water-decomposing capabilities offers the advantage of self-replication. After a single injection, they can exert their effects over an extended period. In recent years, various microorganism preparations with distinct functionalities have been employed in cancer therapy. For example, engineered bacteria modified with Fe_3_O_4_ nanoparticles have been utilized for tumor immunotherapy under magnetic field control [17]. Gene-engineered bacteria for photothermal lysis have been employed to regulate amino acid metabolism, thereby inhibiting tumor growth [18]. Additionally, utilizing photosynthetic activity of algae to generate oxygen helps alleviate the local tumor hypoxic microenvironment, enhancing the efficacy of photodynamic therapy [19]. Compared to conventional drug carriers, microorganisms are capable of providing targeted propulsion through tropism, enabling penetration into tumor tissues. They can also respond to the tumor microenvironment, facilitating individualized proliferation, metabolism, or secretion of specific cell factors [20–22]. Furthermore, due to the diverse array of microbial species in the natural world, each possessing a wide range of functional characteristics, this provides ample options for addressing various tumor treatment requirements and allowing for personalized therapeutic approaches.

**Scheme 1.**
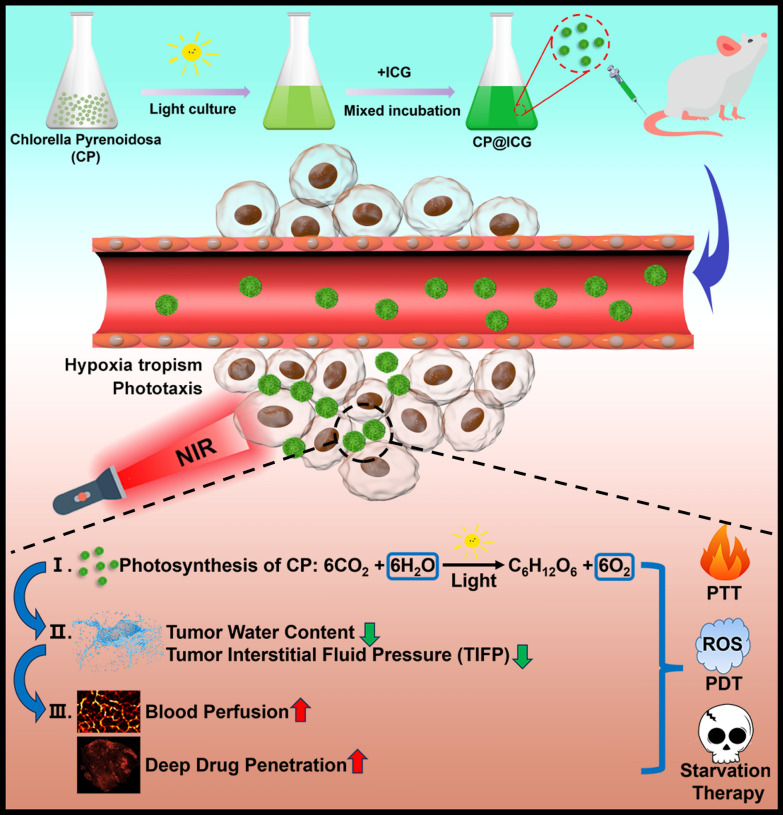
Synthesis and Mechanism of Living Drug Carrier CP@ICG. CP@ICG is obtained by loading indocyanine green (ICG) onto the surface of chlorella pyrenoidosa (CP). Upon intravenous injection, CP@ICG actively accumulates at the tumor site through both phototaxis and hypoxic tropism. Through photosynthesis, CP degrades excess water within the tumor, reducing tumor interstitial fluid pressure (TIFP), thereby increasing local tumor blood perfusion and enhancing deep drug penetration. CP generates oxygen under light exposure, alleviating the tumor's hypoxic microenvironment and boosting the PDT effect of ICG. Under NIR light stimulation, ICG converts light energy into heat, achieving PTT. The proliferation of CP depletes glucose within the tumor, facilitating starvation therapy

This study employed *Chlorella Pyrenoidosa* (CP), a microalga possessing photosynthetic water-splitting capabilities, as an active carrier. Indocyanine green (ICG), a photosensitizer, was loaded onto CP to form the CP@ICG composite therapeutic system (Scheme 1). CP exhibits hypoxia tropism and phototropism, allowing it to actively target tumor sites. Upon exposure to 808 nm near-infrared light, ICG generates excellent photothermal and photodynamic therapeutic effects. Furthermore, under irradiation with 660 nm near-infrared light, CP employs photosynthesis to decompose excess water within the tumor interstitium and produce oxygen, thereby reducing interstitial fluid pressure, enhancing deep-seated penetration, alleviating tumor hypoxia, and augmenting photodynamic therapy effectiveness. Concurrently, CP proliferates locally within the tumor, continuously consuming glucose within the tumor, achieving starvation therapy. Animal experiments demonstrate that CP@ICG can form numerous pores within mouse tumor tissues, significantly enhancing intratumoral blood perfusion. Consequently, this markedly increases the accumulation and penetration of the chemotherapeutic agent DOX within the tumor. This research not only effectively addresses the issue of deep-seated drug delivery in tumor regions but also achieves a combined effect of photothermal/photodynamic/starvation therapy. It presents a new paradigm for the application of microorganisms in tumor therapy.

## Materials

### Materials

*Chlorella Pyrenoidosa* (CP) was purchased from Freshwater Algae Culture Collection at the Institute of Hydrobiology (Wuhan, China). Indocyanine green (ICG) was purchased from Meilun Bio-technology Co., Ltd (Dalian, China). 1,3-diphenylisobenzofuran (DPBF) was purchased from Aladdin Bio-Chem Technology Co., Ltd (Shanghai, China). 2′, 7′-dichlorofluorescin diacetate (DCFH-DA) reactive oxygen species assay kit was purchased from Beyotime Institute of Biotechnology (Shanghai, China). Ultrapure water was prepared by Milli-Q Ultrapure Water Purification System (Millipore, Inc., USA). HeLa and U14 tumor cells were purchased from Chinese academy of medical sciences tumor cells bank (Beijing, China). All animals were purchased from Vital River Laboratory Animal Center (Beijing, China). All animal experimental protocols were approved by the Experimental Animal Ethics Committee of Department of Bioengineering, School of Environment and Chemical Engineering, Yanshan University (No. 202102–10).

### Synthesis and characterization of CP@ICG

CP was cultured in BG11 liquid medium at a temperature of 37 °C under an external light source with an intensity of 300 μmol·m^−2^·s^−1^ and a light–dark cycle of 12 h light and 12 h dark. CP in the logarithmic growth phase was collected for centrifugation (6000 rpm, 10 min). The supernatant was discarded and the cells were resuspended in physiological saline. This process was repeated three times. The CP concentration was adjusted to 1 × 10^7^ cells/ml and stored in the dark for later use. Next, 4 mL of CP suspension was mixed with 1 mL of ICG solution at a concentration of 1 mg/mL in a beaker. The mixture was stirred at room temperature in the dark at a speed of 200 rpm for 10 h to allow thorough contact between ICG and CP cells and to load the CP cells with ICG. Then, the sample was repeatedly washed five times by centrifugation (6000 rpm, 10 min) and resuspended in physiological saline to obtain CP@ICG. The concentration of CP@ICG was adjusted to 1 × 10^7^ cells/mL and stored at room temperature in the dark for later use.

### CP viability assay

CP and CP@ICG solutions were collected and the concentration was adjusted to 1 × 10^7^ cells/mL. Equal volumes of the solutions were then aseptically inoculated into sterile BG11 medium for cultivation under the conditions of 37 °C, an external light intensity of 300 μmol·m^−2^·s^−1^ and a light/dark cycle of 12 h each. Cell concentrations in both solutions were recorded over a period of seven days.

### Characterization of hypoxia and phototactic properties

To induce hypoxia conditions, 400 μL of a 0.4 mg/mL glucose solution together with 0.5 KU glucose oxidase and 0.5 KU catalase were added to the lower chamber of the Transwell. As a control group, 400 μL of saline solution were added to the lower chamber of the Transwell. Then, 200 μL of CP@ICG solution with a concentration of 1 × 10^7^ cells/mL was introduced into the upper chamber. After 40 min of static incubation, the CP concentration in the lower chamber solution was calculated.

Equal volumes of CP@ICG were divided into different tubes and sealed with a sealing film. One side of the tubes was wrapped with light-blocking paper to create a dark environment. The tubes were placed horizontally on a platform and uniform lighting was provided from above. After 8 h of irradiation, the light-blocking paper was carefully removed to avoid fluid disturbances and images were taken to record CP aggregation in each group.

### Photosynthetic oxygen production and water splitting performance of CP@ICG

CP suspension with a concentration of 1 × 10^7^ cells/mL was divided into two small beakers in equal volumes. One group was illuminated with white light as a source for photosynthesis, while the other group was exposed to 660 nm near-infrared light with a power of 0.5 W for photosynthesis. Both groups were irradiated for 10 min and the dissolved oxygen content in the solution was measured and recorded every 1 min with a dissolved oxygen meter (JPBJ-611Y, REX). Then, equal volumes of CP suspension (1 × 10^7^ cells/ml), CP@ICG composite system solution and ultrapure water were placed in separate beakers. All were exposed to 0.5 W 660 nm NIR light as a source for photosynthesis. They were continuously irradiated for three minutes and the dissolved oxygen content in the water was measured every 30 s and recorded with a dissolved oxygen meter.

### Characterization of the ability of CP@ICG to generate singlet oxygen

1 mg/mL solution of DPBF was used as a singlet oxygen detection reagent [23]. 50 L of DPBF was added separately to 2 mL solutions containing 43 μg/mL ICG and 1 × 10^7^ cells/mL CP@ICG. They were then subjected to different light treatments. One group was exposed to 0.5 W 660 nm NIR light for 8 min, another to 2 W 808 nm NIR light for 8 min, and the last group was exposed to alternating 0.5 W 660 nm and 2 W 808 nm NIR light for 8 min. Absorbance curves ranging from 350 to 550 nm were recorded and measured every two minutes using a microplate reader (SpectraMax M2, Molecular Devices).

### Photothermal performance of CP@ICG

Take 1 ml of CP@ICG solution with a concentration of 1 × 10^7^ cells/mL. Illuminate separately with 1.5W and 2W 808 nm NIR light for 5 min. Then take another 1 mL of CP@ICG solution with a concentration of 1 × 10^7^ cells/mL and illuminate it with 2 W 808 nm NIR for 5 min. Measure and record the solution temperature every minute using an infrared thermal imaging camera (InfraTec VarioCAM).

Take 1 mL of CP@ICG solution with a concentration of 1 × 10^7^ cells/mL and subject it to various light treatments: 1) Illumination with 0.5 W NIR light at 660 nm for 5 min; 2) Simultaneous illumination with 2W of 808 nm and 0.5W of 660 nm NIR light for 5 min; 3) Illuminate with 2W 808 nm NIR light for 5 min. Use ultrapure water as a blank control and illuminate with 2W 808 nm NIR light for 5 min. Measure and record the temperature changes of the solutions every minute with an infrared thermal imaging camera.

Take 1 mL of CP solution with a concentration of 1 × 10^7^ cells/mL, 1 mL of CP@ICG solution with a concentration of 1 × 10^7^ cells/mL, 1 mL of ICG solution with a concentration of 43 μg/mL and 1 mL of ultra-pure solution water. Apply 2W 808 nm NIR light to each group for 5 min. Measure and record the temperature of each solution every minute.

### Cell experiment

The solutions of CP and CP@ICG with a concentration of 1 × 10^7^ cells/mL were mixed with HeLa cells and co-cultured for 24 h (under conditions of 37 ℃ and 5% CO_2_). Cell viability was then assessed using the MTT assay. Viability was also observed using FDA/PI staining and inverted fluorescence microscopy (MF52, Mshot). DCFH-DA was used as a probe to observe and record the generation of ROS in differently treated cells.

### Animal experiment

The experimental animals were purchased from Liaoning Changsheng Biotechnology Co., Ltd. All animal experimental procedures were in accordance with the requirements of the Animal Experimentation Ethics Committee of Yanshan University. 200 μL of U14 mouse cervical cancer cells at a concentration of 2.0 × 10^6^ cells/mL were subcutaneously inoculated into the right hind leg root of the mice. When the tumor volume reached 100 mm^3^, all tumor-bearing mice were randomly divided into treatment groups as follows: (1) Saline group (Control), (2) After the injection of CP@ICG, the next day, irradiation was performed with a 0.5 W 660 nm laser for 3 min, followed by a 2-min irradiation with a 2 W 808 nm laser. (CP@ICG + 660 nm + 808 nm), (3) After injection of CP@ICG, the next day, irradiation was performed with a 2 W 808 nm laser for 2 min (CP@ICG + 808 nm), (4) After injection of CP, the next day, irradiation was performed with a 0.5 W 660 nm laser for 3 min (CP + 660 nm), (5) After injection of CP@ICG, the next day, doxorubicin (DOX) was injected, followed by a 3-min irradiation with a 0.5 W 660 nm laser. (CP@ICG + DOX + 660 nm), (6) Only injection of DOX (DOX). All groups were administered via intravenous injection. During the treatment period, the weight and tumor volume of each group of mice were recorded every two days. In addition, in the CP@ICG treatment groups, the local temperature changes of the tumor after irradiation with 808 nm NIR light were recorded and photographed with an infrared thermal imaging camera. After treatment is completed, collect blood from the mice to test various blood routine indicators as well as liver and kidney function indicators. Analyze the influence of CP@ICG on the normal functions of the mice. Dissect the mice's tumors, measure the tumor volume, and perform HE staining on sections of the heart, liver, spleen, lung, kidney, and tumor. Tumor tissues from mice were collected and sectioned. The intratumoral distribution of CP or DOX was observed using laser confocal microscopy (Nikon AX).

Two groups of tumor-bearing mice were selected, one group was injected with saline and the other group was injected with CP@ICG and irradiated with 660 nm NIR light. The changes in local tumor blood perfusion in the two groups of mice were compared using a photoacoustic imaging system.

Six tumor-bearing mice were randomly selected. Each mouse was injected with 200 μL of CP@ICG solution. The intratumoral pressure was measured every other day using a manometer. Subsequently, each mouse was exposed to 0.5 W 660 nm laser for 3 min at the tumor site. The intratumoral pressure was measured again thereafter.

Twelve tumor-bearing mice were randomly selected and divided into two groups. Each mouse was injected with 200 μL of CP@ICG solution. Every other day, the mice were exposed to 0.5 W 660 nm NIR light for 3 min at the tumor site. The control group of mice did not receive light exposure. After two weeks of treatment, both groups of mice were euthanized. Tumors were dissected, weighed, and then placed in an oven for drying. After complete evaporation of water in tumor, the tumors were weighed again. The change in tumor mass before and after drying represented the intratumoral water content.

Healthy mouse blood was collected into EDTA anticoagulant tubes and mixed thoroughly to prevent blood clotting. The blood was then transferred to centrifuge tubes and red blood cells were collected after centrifugation. The red blood cells were diluted with PBS to obtain a 2 v% red blood cell solution. CP@ICG was diluted with PBS to concentrations of 1 × 10^5^ cells/mL, 1 × 10^6^ cells/mL and 1 × 10^7^ cells/mL. 1 mL of CP@ICG solution at various concentrations was mixed with 1 mL of 2% red blood cells. 1 mL of 2 v% red blood cells mixed with deionized water was used as positive control group and 1 mL 2 v% red blood cells mixed with PBS was used as negative control group. Three parallel experiments were performed for each group. All groups were placed in 37 ℃ for 30 min. After incubation, centrifugation was performed, the supernatant was collected, and its absorbance was measured at 570 nm to calculate the hemolysis rate.

### Statistical analysis

All the data are expressed as mean ± SD. The ANOVA analysis was performed using at least three repetitive experiments for each group. The statistical significance between different groups was considered significant for *P < 0.05 and **P < 0.01.

## Results and discussion

### Synthesis and Characterization

**Fig. 1:**
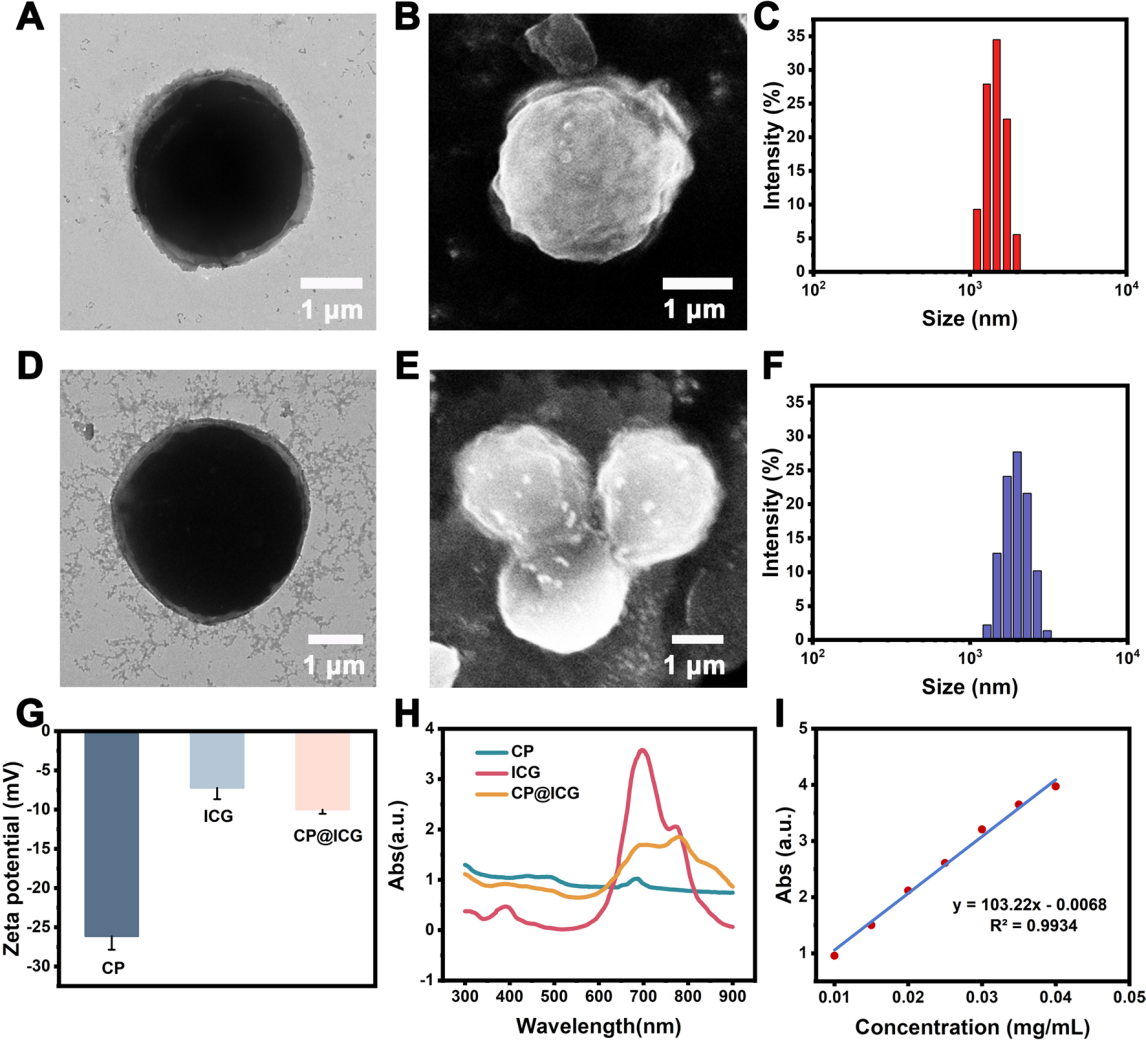
Synthesis and characterization of CP@ICG. **A** TEM image of CP. **B** SEM image of CP. **C** Particle size distribution of CP. **D** TEM image of CP@ICG. **E** SEM image of CP@ICG. **F** Particle size distribution of CP@ICG. **G** Zeta potentials of CP, ICG, and CP@ICG. **H** UV–visible absorption spectra of CP, ICG, and CP@ICG. **I** Standard curve of ICG concentration vs. absorbance

In the characterization via transmission electron microscopy (TEM), scanning electron microscopy (SEM), and conventional optical microscopy, CP exhibited a regular spherical structure (Fig. 1A, B). The cytoplasm appeared green (Additional file 1: Fig. S1), and it was enveloped by a cell wall structure. CP has a harder cell wall, which is mainly composed of cellulose, forming a unique cell wall microfibril structure. These microfibrils form a fine mesh structure, which provides conditions for ICG binding. The particle size analysis (Fig. 1C) indicated an average size of 2.23 ± 0.13 μm for CP. Following external modification and loading with ICG, the resulting CP@ICG exhibited a similar regular spherical structure (Fig. 1D, E), with an average size of 2.25 ± 0.15 μm (Fig. 1F). Notably, CP@ICG showed no significant alterations in morphology or size compared to CP. As shown in Fig. 1G, the zeta potentials of CP, ICG, and CP@ICG were measured as -26.13 ± 1.72 mV, -7.21 ± 1.45 mV, and -10.00 ± 0.52 mV, respectively. Due to the relatively low potential of ICG loaded on the surface of CP, which covers a portion of the charges, the Zeta potential of CP@ICG was significantly reduced. To further confirm the successful synthesis of CP@ICG, UV–Visible absorption spectroscopy was performed on the CP@ICG aqueous solution. As shown in Fig. 1H, the absorption curve of CP@ICG exhibits the characteristic absorption peak of ICG at 780 nm. These results collectively affirm the successful loading of ICG. To quantify the concentration of ICG in the CP@ICG, we conducted measurements and constructed a standard curve relating ICG concentration to absorbance, as depicted in Fig. 1I. Through calculations, it was determined that the concentration of ICG in the CP@ICG was 43 μg/mL.

### Phototaxis and Hypoxia Tropism of CP@ICG

**Fig. 2:**
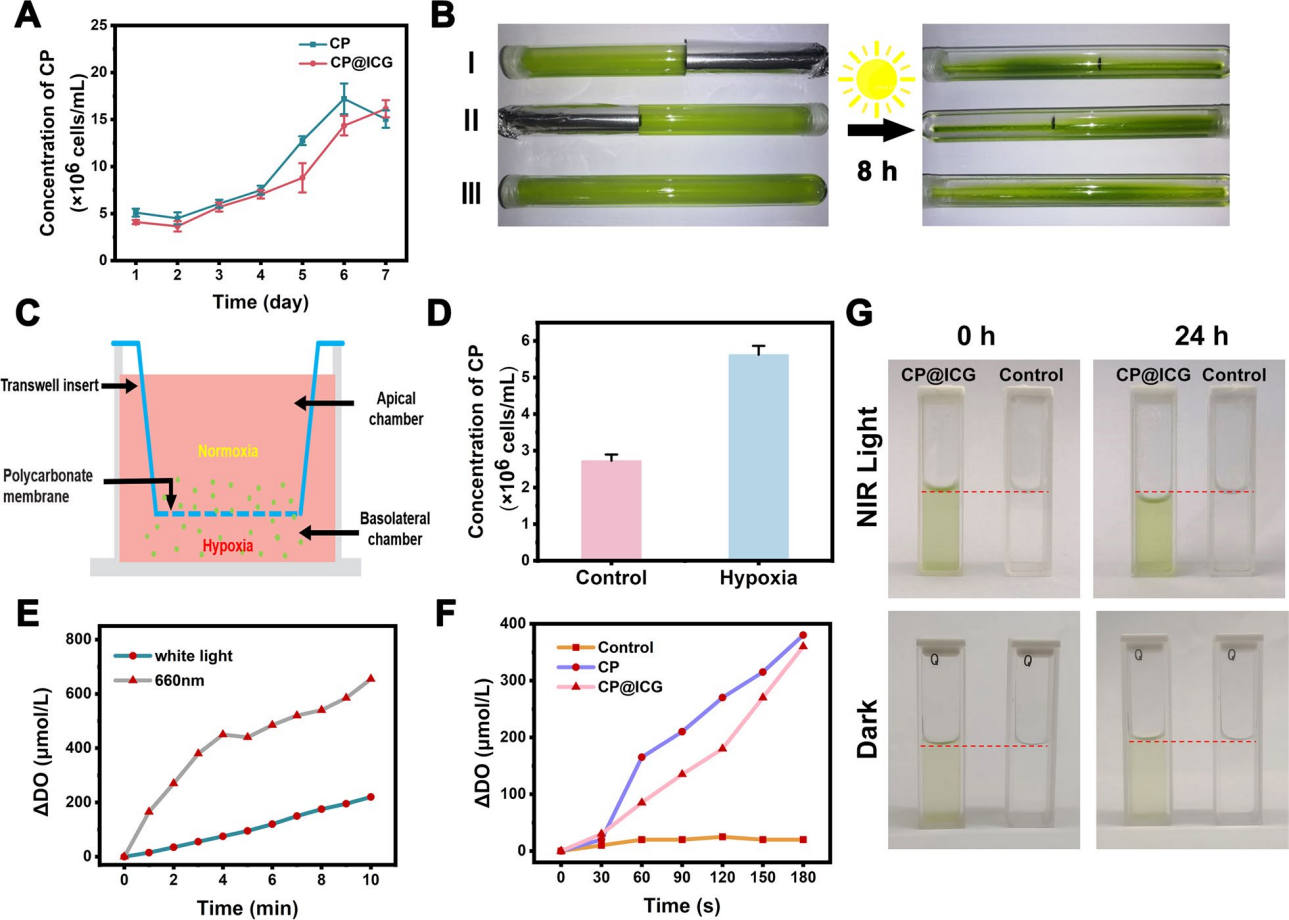
Tropism and Photosynthetic Oxygen Evolution.** A** Proliferation of CP and CP@ICG under 660 nm NIR light irradiation. **B** Phototactic characterization of CP. **C** Schematic representation of CP's hypoxia tropism characterized by Transwell experiment. **D** Concentration of CP under hypoxic conditions in the Transwell experiment. **E** Oxygen evolution of CP@ICG under white light and 660 nm NIR light irradiation. **F** Comparison of oxygen evolution between CP and CP@ICG under 660 nm NIR light irradiation. **G** Water decomposition effect of CP@ICG and Control group (ultrapure water) under 660 nm NIR light irradiation or dark condition

Maintaining the physiological activity of CP is a prerequisite for achieving tumor targeting and treatment with CP@ICG. Therefore, we conducted a one-week growth kinetic monitoring. As shown in Fig. 2A, CP@ICG exhibited a growth trend similar to CP, indicating that its biological activity was not affected by the presence of ICG. This result ensures the functional integrity of CP@ICG for subsequent treatments.

The phototactic behavior of CP@ICG was examined by allowing CP@ICG to undergo static cultivation in glass test tubes, with some of the test tubes being partially shaded. As depicted in Fig. 2B, after 8 h of static light incubation, due to gravity, most of the CP@ICG was found to be settled at the bottom of the test tube. In comparison to the shaded portion, it was observed that the solution in the unshaded area appeared to be a deeper shade of green, indicating a higher concentration of CP in that region. This phenomenon was attributed to two contributing factors: on one hand, owing to the abundant chlorophyll content in CP, it could efficiently engage in photosynthesis, thereby providing itself with ample organic substances and energy. Consequently, CP tended to thrive in environments with abundant light, displaying positive phototaxis. On the other hand, sufficient light stimulation promoted the photosynthetic autotrophic reproduction of CP, leading to an increase in concentration and a deepening of the solution's green color. To further confirm that the concentration variation of CP in test tubes I and II was not solely due to light-induced proliferation, but also related to its phototactic behavior, the test tube without any shading (No. III) was used as a control group. In comparison to the color of the solution in the control group test tube, it was observed that the unshaded areas in test tubes I and II exhibited a deeper color, indicating a higher concentration of CP. Taken together, these results confirmed the strong phototactic behavior of CP@ICG.

Furthermore, the hypoxia tropism of CP@ICG was assessed through a Transwell experiment, as depicted in Fig. 2C. CP@ICG was introduced into the upper chamber, which was maintained under normoxic conditions, while the lower chamber was subjected to hypoxic conditions. Following a static incubation period of 40 min, it was observed that the concentration of CP@ICG in the lower chamber exceeded that in the upper chamber (Fig. 2D). This observation substantiates the hypoxia tropism of CP@ICG, thereby facilitating its responsive accumulation in the hypoxic tumor microenvironment in vivo [24].

### Photosynthetic oxygen production and water splitting performance of CP@ICG

Under illuminated conditions, CP, as an efficient photosynthetic microorganism, can utilize Photosystem II (PSII) to cleave water into protons and electrons, subsequently releasing oxygen [25]. As shown in Fig. 2E, compared to white light, CP exhibited enhanced photosynthetic oxygen production under 0.5 W 660 nm NIR light exposure. The oxygen production rate reached 655 μmol/L in 10 min, approximately three times higher than that under white light conditions. Additionally, CP@ICG demonstrated robust photosynthetic oxygen production capability (Fig. 2F). After 3 min of exposure to 0.5 W 660 nm near-infrared light, the oxygen production rate reached 360 μmol/L. This result further confirms that the loading of ICG did not adversely affect the photosynthetic activity of CP. Figure 2G demonstrates the effective photosynthetic water-splitting capability of CP@ICG. These findings collectively indicate that CP@ICG can undergo efficient photosynthesis under 0.5 W 660 nm NIR light exposure. On one hand, CP@ICG can decompose excess water within the tumor tissue, thereby reducing interstitial fluid pressure, enhancing local blood perfusion, and facilitating the transportation of more drugs into the tumor. On the other hand, the oxygen released during water decomposition can alleviate the local hypoxic tumor microenvironment, providing an ample oxygen source for ICG-mediated photodynamic therapy.

### Singlet oxygen generation ability of CP@ICG

**Fig. 3:**
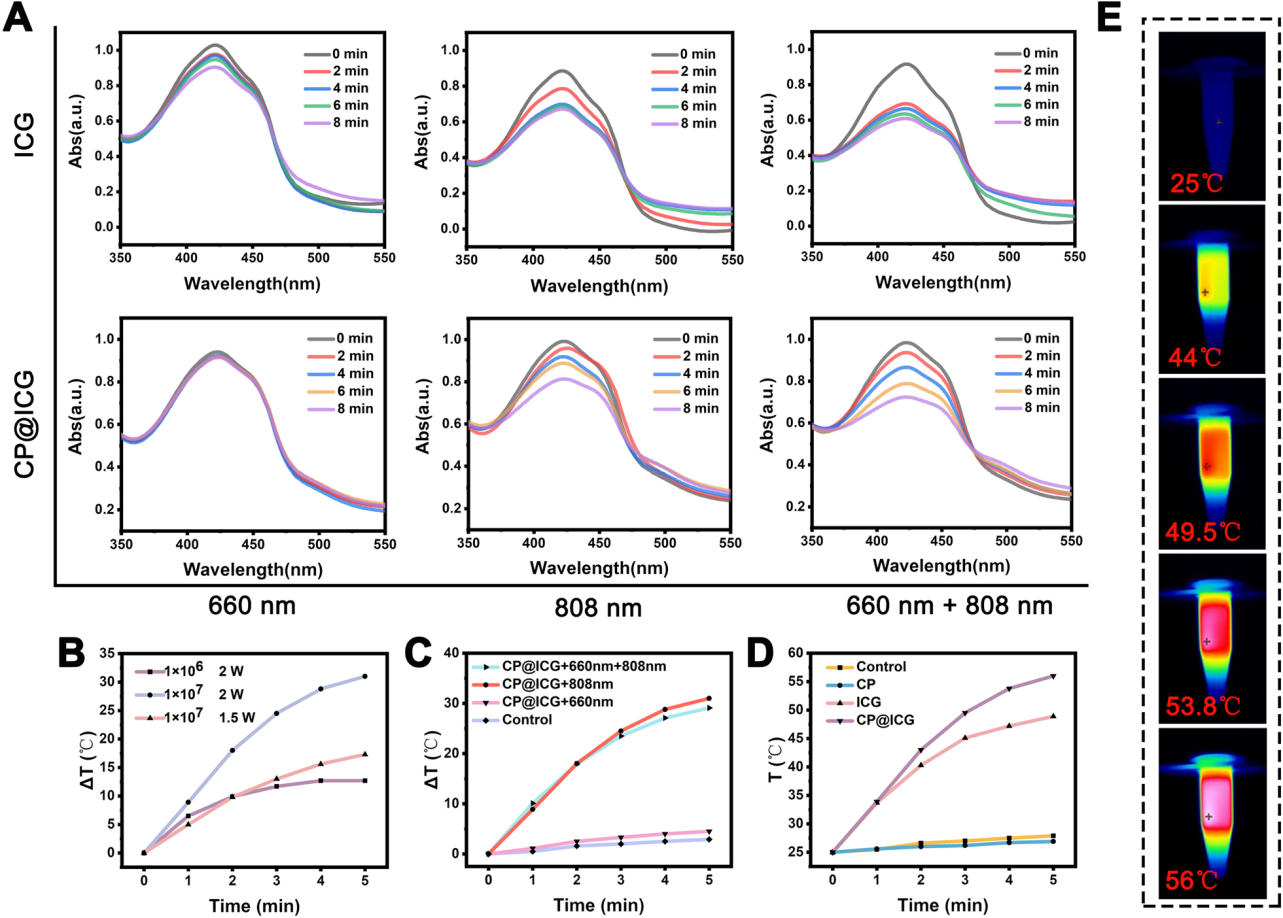
The photodynamic and photothermal performance of CP@ICG. **A** ROS generation of ICG and CP@ICG under different light conditions (660 nm, 808 nm, 660 nm + 808 nm), with DPBF as the ROS detection probe. **B** Influence of different CP@ICG concentrations and different NIR (808 nm) power densities on photothermal effect. **C** Influence of different lighting conditions on photothermal effect. **D** Photothermal effects of different solutions (CP, ICG, CP@ICG) under 808 nm NIR light irradiation. **E** Thermal images of CP@ICG under 808 nm NIR light irradiation

To ascertain the photodynamic therapy (PDT) efficacy of CP@ICG, we employed the DPBF probe to investigate the singlet oxygen generation capabilities of ICG and CP@ICG under varying light irradiation conditions. As illustrated in Fig. 3A, the capacity for active oxygen production by ICG and CP@ICG under 660 nm NIR light stimulation was significantly lower compared to that under 808 nm and 660 nm + 808 nm stimulation. This phenomenon can be attributed to the lower light absorption capacity of ICG at 660 nm in contrast to 808 nm. Additionally, under 660 nm NIR light stimulation, the production of singlet oxygen in CP@ICG was lower compared to ICG. This is attributed to the substantial chlorophyll content in CP, which exhibits a robust absorption capacity for 660 nm NIR light (Fig. 1H). Consequently, this diminishes ICG's absorption and utilization of light, leading to reduced singlet oxygen generation. Compared with 808 nm irradiation alone, the singlet oxygen generation capacity of CP@ICG was significantly enhanced when 660 nm + 808 nm NIR light was co-stimulated. This is due to the photosynthetic generation of oxygen by chlorophyll in CP under 660 nm NIR light exposure, providing an increased oxygen source for ICG's photodynamic process, thereby promoting singlet oxygen production. In summary, these results demonstrate that CP@ICG possesses excellent singlet oxygen generation ability, meeting the conditions required for photodynamic therapy. Furthermore, the photosynthetic oxygen production process of CP can further enhance the effectiveness of photodynamic therapy.

### Photothermal performance of CP@ICG

In addition to serving as a photosensitizer for photodynamic therapy, ICG also exhibits remarkable photothermal conversion performance, efficiently transforming light energy into heat, which can be employed for photothermal therapy of tumors [26]. As shown in Fig. 3B, with increasing concentration and NIR light power, CP@ICG's heating effect was significantly enhanced. When the concentration of CP@ICG was 1 × 10^7^ cells/mL and the NIR light power is 2 W, the temperature of the CP@ICG solution increased by 31 ℃ after 5 min, fully meeting the requirements for photothermal therapy. Furthermore, the temperature changes in the CP@ICG solution under NIR light irradiation of different wavelengths were investigated. As observed in Fig. 3C, sole irradiation with 660 nm NIR light did not cause a significant change in the temperature of the CP@ICG solution, while 808 nm and 660 nm + 808 nm could notably elevate the solution temperature. The underlying mechanism lies in the capacity of 660 nm NIR light to prompt the transition of ICG molecules from their ground state to an excited state. As these molecules revert back to their ground state, the discharged energy is conveyed to adjacent oxygen molecules. This process culminates in the production of singlet oxygen, ultimately effectuating the desired outcome of photodynamic therapy [27]. In contrast, 808 nm NIR light not only induces photodynamic effects in ICG but also converts the absorbed light energy into heat, thereby realizing photothermal therapy [28].

Figure 3D illustrated the temperature increase in different solutions under 808 nm NIR light irradiation. It was evident from the figure that CP@ICG exhibited the most efficient photothermal effect, surpassing even ICG. This phenomenon was inferred to be attributed to the binding of CP and ICG, which enhanced the system's absorption of NIR light, consequently increasing the overall photothermal conversion capability. Figure 3E presented the infrared thermographic image of the CP@ICG solution, consistent with the temperature variation curve. All the results corroborated the outstanding photothermal performance of CP@ICG.

### Analysis of intracellular ROS production

**Fig. 4:**
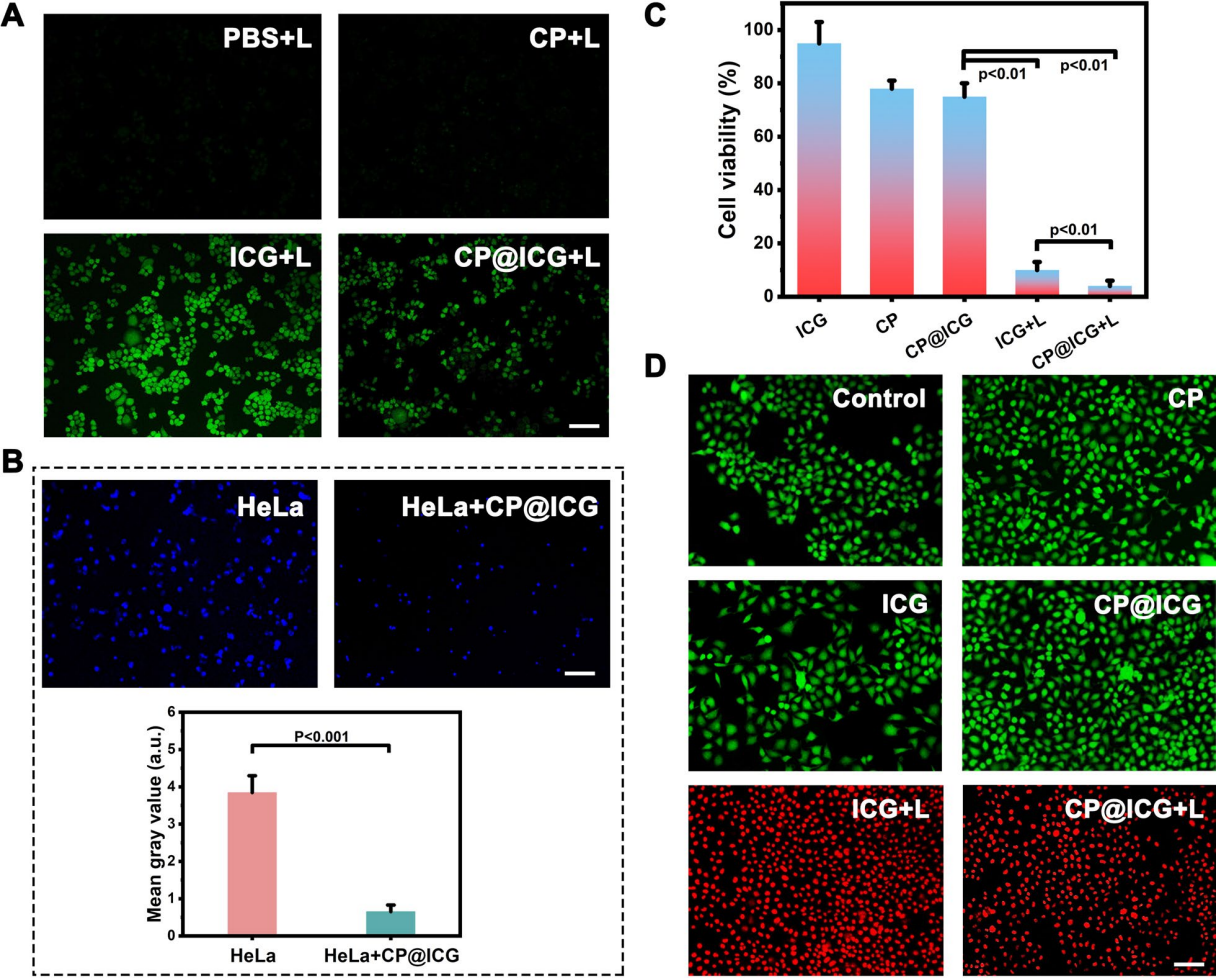
Cell experiments. **A** Detection of intracellular ROS production under different treatment conditions using DCFH-DA as a probe. **B** The effect of CP@ICG on HeLa cell proliferation, with cell nuclei labeled in Hoechst (blue) and quantitatively analyzed using Image J software. **C** Cell viability under different treatment conditions. **D** Live/dead cell staining under different treatment conditions, with FDA in green and PI in red. Scale bar: 100 µm. ("L" represents NIR irradiation, 0.5W 660 nm + 2W 808 nm, 5 min)

ROS is a byproduct of normal cellular metabolism with an extremely short lifespan, and it exerts no significant toxic effects on cells [29]. However, artificially inducing an excessive production of ROS within cells can lead to apoptosis or even necrosis through oxidative stress reactions [30]. As a photosensitizer, ICG can generate singlet oxygen under NIR light exposure, thus artificially promoting an overload of intracellular ROS. As shown in Fig. 4A, it is evident that both the PBS and CP groups did not exhibit any green fluorescence after exposure to 808 nm laser. In contrast, when co-incubated with HeLa cells and subjected to NIR light, ICG produced a strong green fluorescence signal, indicating the generation of a substantial amount of ROS. The CP@ICG also exhibited a green fluorescence signal after irradiation with 808 nm laser. However, the fluorescence intensity was significantly lower compared to the ICG group. The reason for this was that ICG is loaded onto the surface of CP and binds with CP cells. Due to the larger size of CP cells, they were less likely to be taken up by tumor cells. During the experiment, with the washing steps, ICG was washed away along with the unabsorbed CP, thereby reducing the fluorescence intensity of the solution. Some ICG detached from the surface of CP and was taken up by HeLa cells, resulting in a weaker intracellular green fluorescence.

### Anti-tumor effect of CP@ICG In Vitro

Cancer cells primarily rely on aerobic glycolysis to meet their energy and proliferation demands, the process known as the Warburg effect [31]. Targeting this metabolic characteristic, tumor cell energy supply can be disrupted by depleting glucose, thereby inducing tumor cell death [32], which is called starvation therapy. The results in Additional file 1: Fig. S2 demonstrate that the presence of glucose promoted the proliferation of CP. Therefore, CP can serve as a glucose competitor, inhibiting tumor cell growth through starvation therapy. To gain further insights into the impact of *Chlorella pyrenoidosa* on tumor cell proliferation, CP was co-cultured with HeLa cells, followed by nuclear staining of HeLa cells using the Hoechst nuclear dye. The results were depicted in Fig. 4B. The fluorescence in the co-culture group of HeLa cells with CP was significantly attenuated compared to the group of HeLa cells cultured alone, with hardly any noticeable proliferation observed. Fluorescence quantification analysis using Image J software indicated a substantial disparity in fluorescence intensity between the two groups. The possible reason is that CP consumes nutrients in the environment, especially glucose, during its growth and reproduction. As the main energy source for HeLa cells, the reduction of glucose limits cell activity and proliferation. Therefore, based on the above experimental results, we speculate that CP slows down or inhibits the growth and reproduction of HeLa cells by competing for nutrients, achieving the effect of starvation therapy.

The impact of different treatment modalities on the inhibition of HeLa cell growth was subsequently investigated (Fig. 4C, D). Pure ICG exhibited no pronounced inhibitory effect on HeLa cells. Conversely, both the CP and CP@ICG groups underwent a form of starvation therapy, resulting in a reduction of HeLa cell proliferation without significant induction of apoptosis. As a result, the number of viable cells relative to the control decreased in the CP and CP@ICG groups in Fig. 4C, with no notable apoptosis observed in Fig. 4D for these groups. In contrast, the ICG + L group experienced a substantial decrease in cell viability and a concurrent increase in apoptosis due to the combined effects of PDT and PTT. In comparison to the ICG + L group, the CP@ICG + L group not only amplified the PDT efficacy of ICG through the enhanced oxygenation capability of CP, promoting further ROS generation, but also demonstrated a significant inhibitory effect on HeLa cells under the combined action of PDT, PTT, and starvation therapy.

### Anti-tumor effect of CP@ICG in vivo

**Fig. 5:**
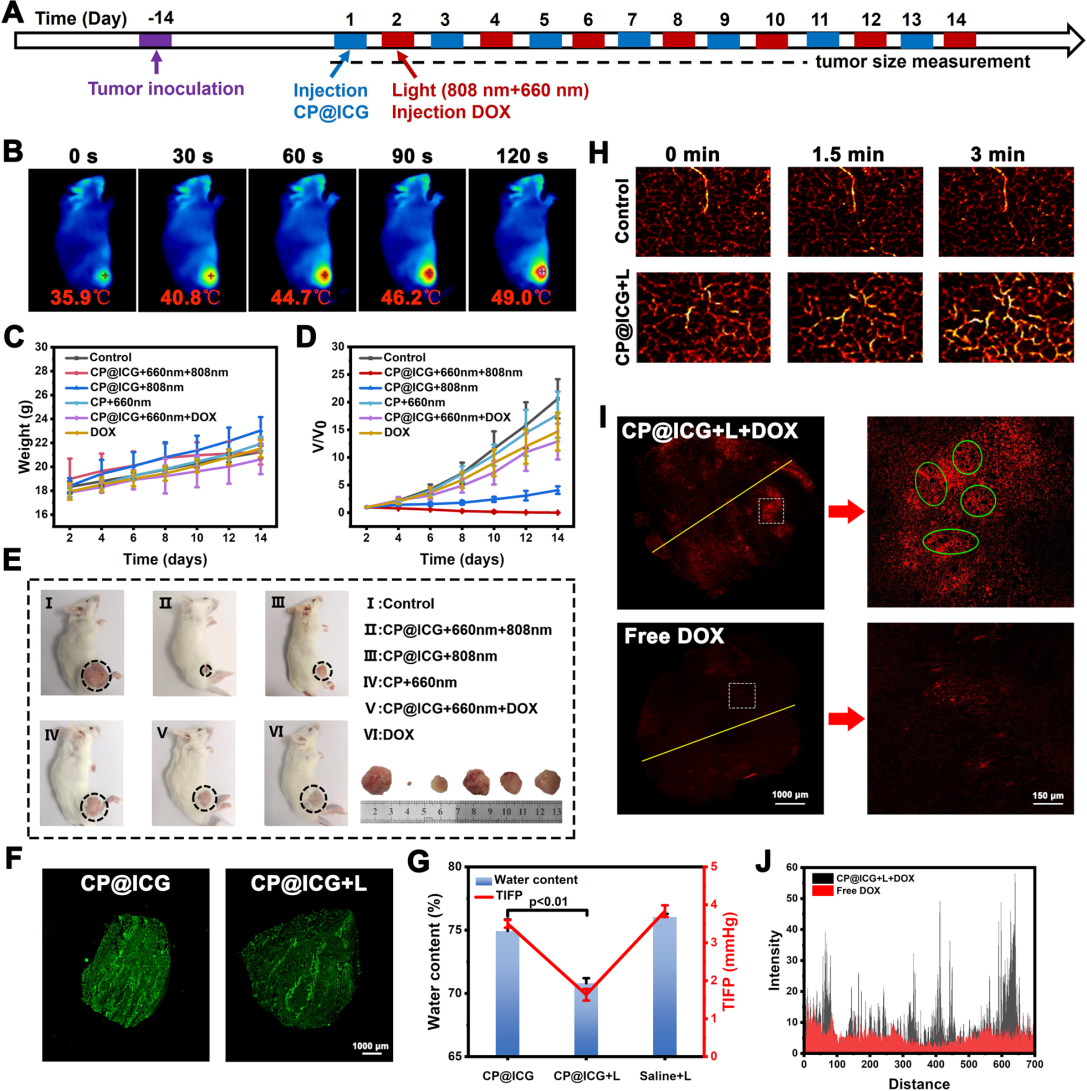
Animal experiments.** A** Schematic diagrams of animal experiment. **B** Thermal imaging of tumor-bearing mice injected with CP@ICG under 808 nm NIR irradiation. **C** Body weight in mice under different treatments. **D** Tumor volume in mice under different treatments. **E** Images of tumors in mice from different treatment groups. **F** Fluorescent tissue section analysis showing the distribution of CP@ICG within the tumor. **G** Changes in tumor tissue water content and tumor interstitial fluid pressure (TIFP) with or without NIR irradiation after intravenous injection of CP@ICG. **H** Photoacoustic imaging of the tumor site after CP@ICG + L treatment, reflecting blood perfusion. **I** Fluorescent tissue section analysis showing the penetration of DOX within the tumor, with significant "hollowing" observed in the enlarged image (green circle). **J** Distribution of fluorescence intensity in the fluorescent tissue section (yellow line)

In the in vivo photothermal experiment, CP@ICG demonstrated photothermal performance comparable to that of ICG. To substantiate its ability to maintain photothermal effects in vivo, the temperature variations at the mouse tumor site during 808 nm laser irradiation were captured and recorded using an infrared camera. As shown in Fig. 5B, with the prolonged irradiation time, the local tumor temperature gradually increased, reaching 49.0 ℃, which was 13.1 ℃ higher than the initial temperature. Since tumor cells are less tolerant to heat compared to normal cells, even mild hyperthermia can be effective in sensitizing tumor cells [33, 34]. Therefore, this temperature is sufficient to induce tumor cell death while avoiding harm to normal cells. These results demonstrated that CP@ICG can achieve excellent photothermal therapy effects in vivo. Furthermore, during the treatment period, all groups of mice showed steady weight gain (Fig. 5C), indicating that the administered therapeutic agents did not interfere with the mice's weight changes, demonstrating good biocompatibility. Figure 5D showed the relative change in tumor volume during the treatment process compared to untreated controls. The control group (saline group) exhibited a consistently rapid growth rate in tumor volume. Compared to the control group, the CP + 660 nm group exhibited a certain inhibitory effect. This can be attributed to the fact that upon reaching the tumor site, CP tends to accumulate in the tumor interstitium due to the rich and viscoelastic nature of tumor cell interstitium. Additionally, CP utilizes nutrients within the tumor site, such as glucose, for its growth and reproduction. This competitive consumption of nutrients within the tumor microenvironment moderately inhibits tumor growth. Thus, the presence of CP demonstrates its role in exerting starvation therapy. The CP@ICG + 808 nm group exhibited a certain level of tumor inhibition. This is attributed to the excellent photothermal effect generated by both CP and ICG under 808 nm laser. Additionally, tumor cells are more heat-sensitive compared to normal cells. Hence, effective eradication and ablation of tumor cells can be achieved through relatively mild photothermal therapy. Of particular note, the CP@ICG + 660 nm + 808 nm group demonstrated the optimal tumor inhibition effect, nearly achieving complete cure. The reason lay in the fact that under 660 nm laser irradiation, CP underwent photosynthesis, leading to the decomposition of water and the generation of oxygen. On one hand, this reduced IFP, thereby enhancing intratumoral drug penetration and delivery efficiency. On the other hand, it alleviated tumor hypoxia, resulting in increased ROS production. Coupled with starvation therapy and photothermal therapy, this ultimately manifested as an outstanding tumor inhibitory effect. In addition, the CP@ICG + 660 nm + DOX treatment group exhibited a superior tumor inhibitory effect compared to the DOX treatment group alone. This could be attributed to the reduction in interstitial fluid pressure (IFP) during treatment, which increased blood perfusion in the tumor site, facilitating drug penetration and enabling a greater amount of DOX to be transported to the tumor site to exert its therapeutic effect. Representative mice and dissected tumor images from each group at the end of the treatment period were shown in Fig. 5E. It was observed that there were differences in tumor growth among the groups after different treatments. The CP@ICG + 660 nm + 808 nm treatment group demonstrated the most significant tumor inhibitory effect, while the tumors in the other groups exhibited varying degrees of growth.

Figure 5F illustrated the distribution of CP in tumors for both the CP@ICG group and CP@ICG + L (CP@ICG + 660 nm) group. As shown in the images, both tumor slices exhibited significant fluorescence signals. This outcome affirmed the excellent tumor targeting and deep penetration capabilities of the drug delivery system based on CP. This property was well validated in Fig. 2D, highlighting CP's tropism for hypoxic environments. Early studies demonstrates that due to their rapid growth, tumors exhibit irregular vascular structures, resulting in a radially hypoxic state within the tumor. As the depth of the tumor increases, the oxygen content gradually decreases [35]. Therefore, the CP-based drug delivery system capitalizes on the hypoxia tropism of CP to navigate towards the hypoxic regions within the tumor, thereby facilitating penetration and drug delivery into the deeper layers of the tumor.

In order to investigate the water depletion effect of CP@ICG in vivo, the tumor water content was assessed across different treatment groups. As shown in Fig. 5G a significant reduction in tumor water content was observed in the group treated with 660 nm NIR light compared to the untreated group. This substantiates the occurrence of photosynthesis by CP@ICG under 660 nm irradiation, thereby depleting local tumor moisture. Our earlier studies have indicated that this reduction in water content leads to a decrease in IFP within the tumor [9, 10, 36]. The results in Fig. 5G similarly confirmed the above theory. After 660 nm NIR light irradiation, a noticeable reduction in IFP was observed, consistent with the change in tumor water content, demonstrating that a reduction in water can lower IFP, thereby increasing local tumor blood perfusion and promoting drug penetration into the tumor. In Fig. 5H, the photoacoustic images depicted the changes in local tumor blood perfusion over time. In the Control group, there was no significant alteration observed in the hemoglobin signal under 660 nm laser irradiation. In contrast, the hemoglobin signal in the CP@ICG group gradually intensified with the prolonged duration of laser irradiation, indicating an augmented blood perfusion. The results indicated that, following stimulation by 660 nm NIR light, CP@ICG facilitated local tumor blood perfusion by reducing IFP through its water-depleting effect.

In Fig. 5I, J it was demonstrated that the treatment with CP@ICG + L significantly enhanced the accumulation of chemotherapy drug DOX at the tumor site. Compared to free DOX, the tumor's internal drug fluorescence intensity notably increased in the CP@ICG + L + DOX group, affirming the effective enhancement of DOX accumulation and deep penetration within the tumor due to the prior treatment with CP@ICG + L. It's worth noting that in the localized magnification from Fig. 5I a substantial number of "hollowing" (highlighted in green circles) were clearly observed within the tumor tissue. It is speculated that this phenomenon may be attributed to the gas generated during the photosynthesis-induced water depletion process by the CP, leading to a localized "aerating" effect. The appearance of these cavities also provided excellent pathways for blood perfusion and deep drug penetration.

### Biological safety assessment

**Fig. 6:**
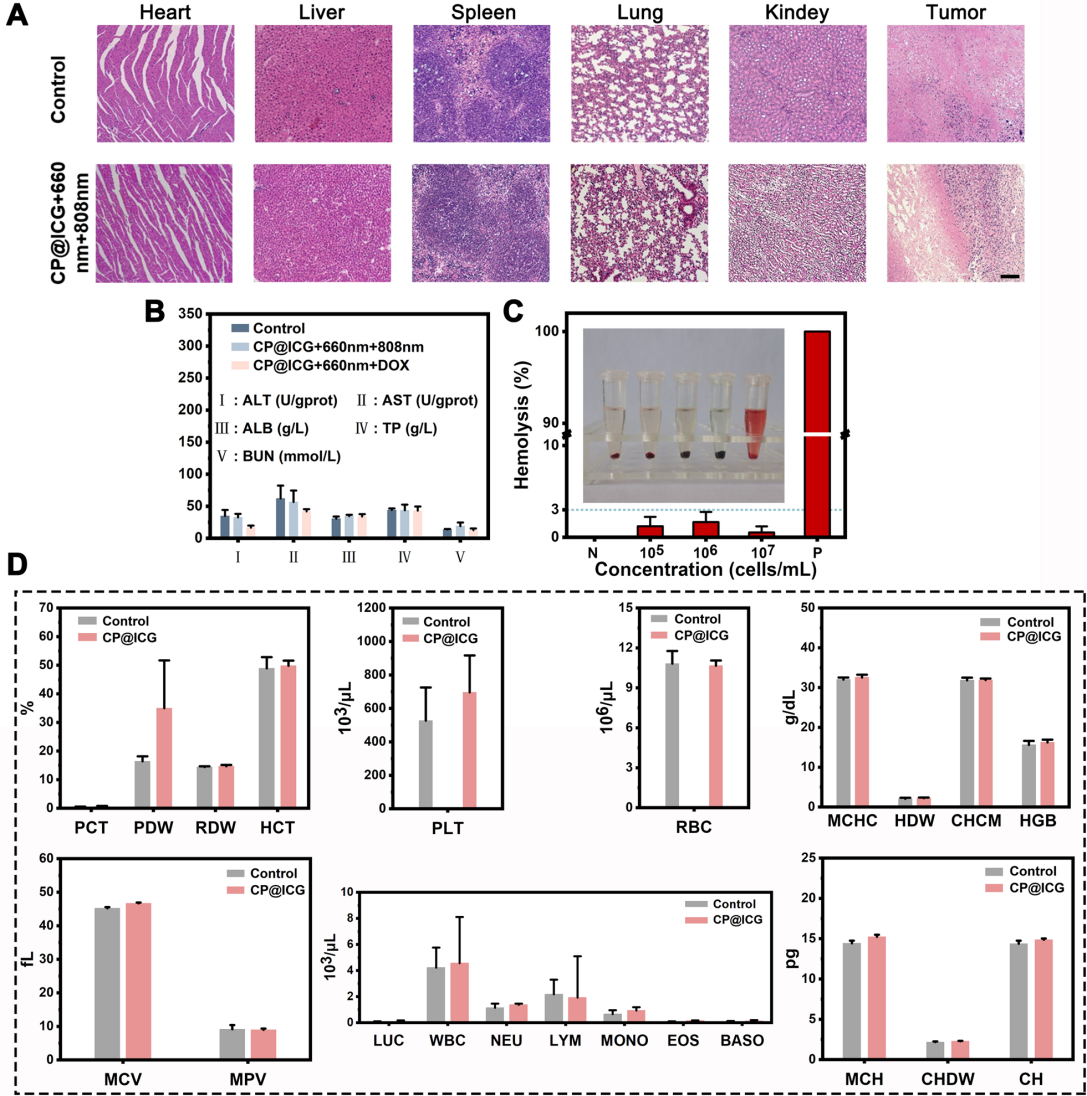
Biosafety assessment. **A** HE-stained images of various tissues in mice after treatment completion. Scale bar: 100 µm. **B** Differences of liver and kidney indicators in mice under different treatments. **C** In vitro hemolysis assay of CP@ICG. **D** Blood indicators in mice after CP@ICG treatment

The results of hematoxylin and eosin (HE) staining are presented in Fig. 6A. Slices of heart, liver, spleen, lung, and kidney from the CP@ICG + 660 nm + 808 nm treatment group showed no significant differences compared to the control group, and both groups did not exhibit apparent damage. However, notable differences were observed in the tumor HE-stained sections between the two groups. Compared to the undamaged control group, clear traces of tissue damage were evident in the CP@ICG + 660 nm + 808 nm treatment group. This outcome substantiates that the CP@ICG + 660 nm + 808 nm treatment approach effectively eliminates tumor cells while safeguarding normal tissue, demonstrating good biocompatibility. The kidneys and liver are two vital organs in the body. In order to further assess the biocompatibility of CP@ICG, various liver and kidney indicators were examined in mice. The results of the tests for alanine aminotransferase (ALT), aspartate aminotransferase (AST), albumin (ALB), total protein (TP), and blood urea nitrogen (BUN) are presented in Fig. 6B. Using the values of healthy mice as a reference, it was observed that there were no significant differences in these five indicators between the CP@ICG + 660 nm + 808 nm and CP@ICG + 660 nm + DOX treatment groups and the healthy control group. This suggests that the treatment approach exerted no substantial toxicity or damage to the liver and kidneys.

Figure 6C presents the results of the hemolysis assay. CP@ICG was tested at three concentration gradients: 1 × 10^5^, 1 × 10^6^, and 1 × 10^7^ cells/mL. The positive control group exhibited a vivid red color in the solution, indicating a significant degree of red blood cell hemolysis. In contrast, the supernatant of the negative control group and other experimental groups remained clear, with only faint traces of red. To calculate hemolysis rate accurately, absorbance measurements were taken from the supernatant. The results of hemolysis rate calculations showed that the maximum hemolysis rate in the experimental groups was less than 3%. This indicates that as the concentration of CP@ICG increased, hemolysis did not occur. These results demonstrate that CP@ICG does not induce hemolysis.

In addition, due to the microorganism CP present in the synthesized system CP@ICG, it was imperative to conduct a hematological examination to ascertain whether the therapeutic agent had any impact on the overall health of the mice. Thus, we assessed twenty-two hematological parameters, encompassing the red blood cell system, white blood cell system, and platelet system. As shown in Fig. 6D, there were no significant differences observed in any of the parameters between the drug-injected group and the blank control group, confirming that CP@ICG had no effect on the blood parameters, indicating its excellent biosafety.

## Conclusion

In recent years, the application of microbial preparations in disease treatment has garnered widespread attention. This study utilized *Chlorella pyrenoidosa* as a carrier to design and construct the CP@ICG system, characterized by its simple structure and outstanding functionality. Leveraging CP's hypoxic tropism and photosynthetic oxygen-producing capabilities, the system achieved targeted aggregation at the tumor site. Under dual near-infrared light stimulation (660 nm + 808 nm), CP@ICG not only decomposed excess water within the tumor, reducing interstitial fluid pressure, promoting blood perfusion and deep-seated penetration, but also realized a combined effect of photothermal therapy, photodynamic therapy, and starvation therapy. Most crucially, as a safe therapeutic modality, CP@ICG significantly enhanced the accumulation and penetration of the chemotherapeutic agent DOX within the tumor under near-infrared light stimulation. This holds the promise of serving as a universal pretreatment approach to augment the therapeutic efficacy of drugs. The research on enhancing drug delivery into tumors through photosynthesis in algae provides innovative insights and scientific basis for cancer treatment.

### Supplementary Information

**Additional file 1: Figure S1.** Optical microscope images of CP. **Figure S2.** The influence of Glucose on the proliferation of CP. **Figure S3.** Proliferation of CP under different light irradiation. **Figure S4.** Cell apoptosis test under different treatment conditions.

## Data Availability

The datasets used and/or analysed during the current study are available from the corresponding author on reasonable request.

## References

[1] Wilhelm S (2016). Analysis of nanoparticle delivery to tumours. Nat Rev Mater.

[2] Lin Y (2023). Folate receptor-mediated delivery of Cas9 RNP for enhanced immune checkpoint disruption in cancer cells. Small.

[3] Kara G, Calin GA, Ozpolat B (2022). RNAi-based therapeutics and tumor targeted delivery in cancer. Adv Drug Deliv Rev.

[4] Wang K (2022). Tumor-acidity and bioorthogonal chemistry-mediated on-site size transformation clustered nanosystem to overcome hypoxic resistance and enhance chemoimmunotherapy. ACS Nano.

[5] Xue X (2023). A transformable nanoplatform with multiple therapeutic and immunostimulatory properties for treatment of advanced cancers. Biomaterials.

[6] Chen H (2023). Transcytosis mediated deep tumor penetration for enhanced chemotherapy and immune activation of pancreatic cancer. Adv Funct Mater.

[7] Sun R (2023). Aminopeptidase N-responsive conjugates with tunable charge-reversal properties for highly efficient tumor accumulation and penetration. Angew Chem.

[8] Cong C (2020). "Nano-lymphatic" photocatalytic water-splitting for relieving tumor interstitial fluid pressure and achieving hydrodynamic therapy. Mater Horiz.

[9] He Y (2021). Pyroelectric catalysis-based "nano-lymphatic" reduces tumor interstitial pressure for enhanced penetration and hydrodynamic therapy. ACS Nano.

[10] Fu Y (2022). Decrease in tumor interstitial pressure for enhanced drug intratumoral delivery and synergistic tumor therapy. ACS Nano.

[11] Taleb M (2021). Bifunctional therapeutic peptide assembled nanoparticles exerting improved activities of tumor vessel normalization and immune checkpoint inhibition. Adv Healthc Mater.

[12] Heldin CH (2004). High interstitial fluid pressure—an obstacle in cancer therapy. Nat Rev Cancer.

[13] Ding J (2019). Engineered nanomedicines with enhanced tumor penetration. Nano Today.

[14] Kim J (2022). Microfluidic one-directional interstitial flow generation from cancer to cancer associated fibroblast. Acta Biomater.

[15] Gao X (2017). Reducing interstitial fluid pressure and inhibiting pulmonary metastasis of breast cancer by gelatin modified cationic lipid nanoparticles. ACS Appl Mater Interfaces.

[16] Hao Z (2022). Photocatalysis/enzymolysis-based biomimetic Schottky junction reduces tumor interstitial solid and fluid phases for deep-penetrating tumor therapy. Chem Eng J.

[17] Ma X (2023). Modular-designed engineered bacteria for precision tumor immunotherapy via spatiotemporal manipulation by magnetic field. Nat Commun.

[18] Sun M (2023). Photothermal lysis of engineered bacteria to modulate amino acid metabolism against tumors. Adv Func Mater.

[19] Wang H (2021). Light-controlled oxygen production and collection for sustainable photodynamic therapy in tumor hypoxia. Biomaterials.

[20] Sun R (2022). Bacteria loaded with glucose polymer and photosensitive ICG silicon-nanoparticles for glioblastoma photothermal immunotherapy. Nat Commun.

[21] Savage TM (2023). Chemokines expressed by engineered bacteria recruit and orchestrate antitumor immunity. Sci Adv.

[22] Chen Y (2023). Bacteria-based bioactive materials for cancer imaging and therapy. Adv Drug Deliv Rev.

[23] Jana D, Zhao Y (2022). Strategies for enhancing cancer chemodynamic therapy performance. Exploration (Beijing).

[24] Feng Y (2023). Emerging nanomedicines strategies focused on tumor microenvironment against cancer recurrence and metastasis. Chem Eng J.

[25] Shen JR (2015). The Structure of Photosystem II and the Mechanism of Water Oxidation in Photosynthesis. Annu Rev Plant Biol.

[26] Zheng M (2013). Single-step assembly of DOX/ICG loaded lipid–polymer nanoparticles for highly effective chemo-photothermal combination therapy. ACS Nano.

[27] Yang L (2022). Indocyanine green assembled free oxygen-nanobubbles towards enhanced near-infrared induced photodynamic therapy. Nano Res.

[28] Ding X (2023). A dual heat shock protein down-regulation strategy using PDA/Cu/ICG/R controlled by NIR "Switch" enhances mild-photothermal therapy effect. Adv Healthc Mater.

[29] Lennicke C, Cocheme HM (2021). Redox metabolism: ROS as specific molecular regulators of cell signaling and function. Mol Cell.

[30] An J (2020). ROS-augmented and tumor-microenvironment responsive biodegradable nanoplatform for enhancing chemo-sonodynamic therapy. Biomaterials.

[31] Sun L (2023). Gold nanoparticles inhibit tumor growth via targeting the Warburg effect in a c-Myc-dependent way. Acta Biomater.

[32] Dai L (2022). Multifunctional metal-organic framework-based nanoreactor for starvation/oxidation improved indoleamine 2,3-dioxygenase-blockade tumor immunotherapy. Nat Commun.

[33] Wang Z (2022). Phototheranostic nanoparticles with aggregation-induced emission as a four-modal imaging platform for image-guided photothermal therapy and ferroptosis of tumor cells. Biomaterials.

[34] Xin Q (2023). Tracking tumor heterogeneity and progression with near-infrared II fluorophores. Exploration (Beijing).

[35] Chen Z (2023). Hypoxic microenvironment in cancer: molecular mechanisms and therapeutic interventions. Signal Transduct Target Ther.

[36] Cong C (2020). “Nano-lymphatic” photocatalytic water-splitting for relieving tumor interstitial fluid pressure and achieving hydrodynamic therapy. Mater Horiz.

