# Supplementary material for: Achieving deep intratumoral penetration and multimodal combined therapy for tumor through algal photosynthesis
Source: J Nanobiotechnology. 2024 May 6;22:227. doi: 10.1186/s12951-024-02476-7 (PMC11075286; doi:10.1186/s12951-024-02476-7)
Supplement: Supplementary file 1 — Additional file 1: Figure S1. Optical microscope images of CP. Figure S2. The influence of Glucose on the proliferation of CP. Figure S3. Proliferation of CP under different light irradiation. Figure S4. Cell apoptosis test under different treatment conditions. [file 12951_2024_2476_MOESM1_ESM.docx]

**Additional file 1**

**Achieving Deep Intratumoral Penetration and Multimodal Combined Therapy for Tumor through Algal Photosynthesis**

Xuwu Zhang^1,2,#^, Xinyue Zhang^1,2,#^, Shiqi Liu^1,2^, Weidong Zhang^3^, Liang Dai^3^, Xifa Lan^3^, Desong Wang^1,2^, Wenkang Tu^1,2,^*, Yuchu He^1,2,^*, Dawei Gao^1,2,^*

1. Nano-biotechnology Key Lab of Hebei Province, Yanshan University, No.438 Hebei Street, Qinhuangdao, 066004, P. R. China.

2. Applying Chemistry Key Lab of Hebei Province, Yanshan University, Qinhuangdao, 066004, P. R. China.

3. Department of Pharmacy, The First Hospital of Qinhuangdao, Qinhuangdao, 066004, P. R. China.

***Corresponding author:**

E-mail: wenkang_tu@ysu.edu.cn (Dr. Wenkang Tu);

hychu@ysu.edu.cn (Prof. Yuchu He)

dwgao@ysu.edu.cn (Prof. Dawei Gao)

Figure S1


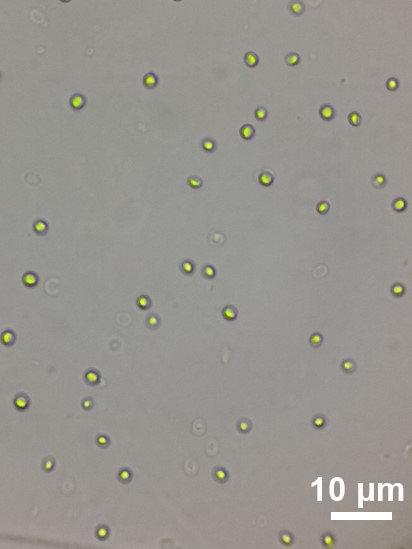


**Figure S1.** Optical microscope images of CP.

Figure S2


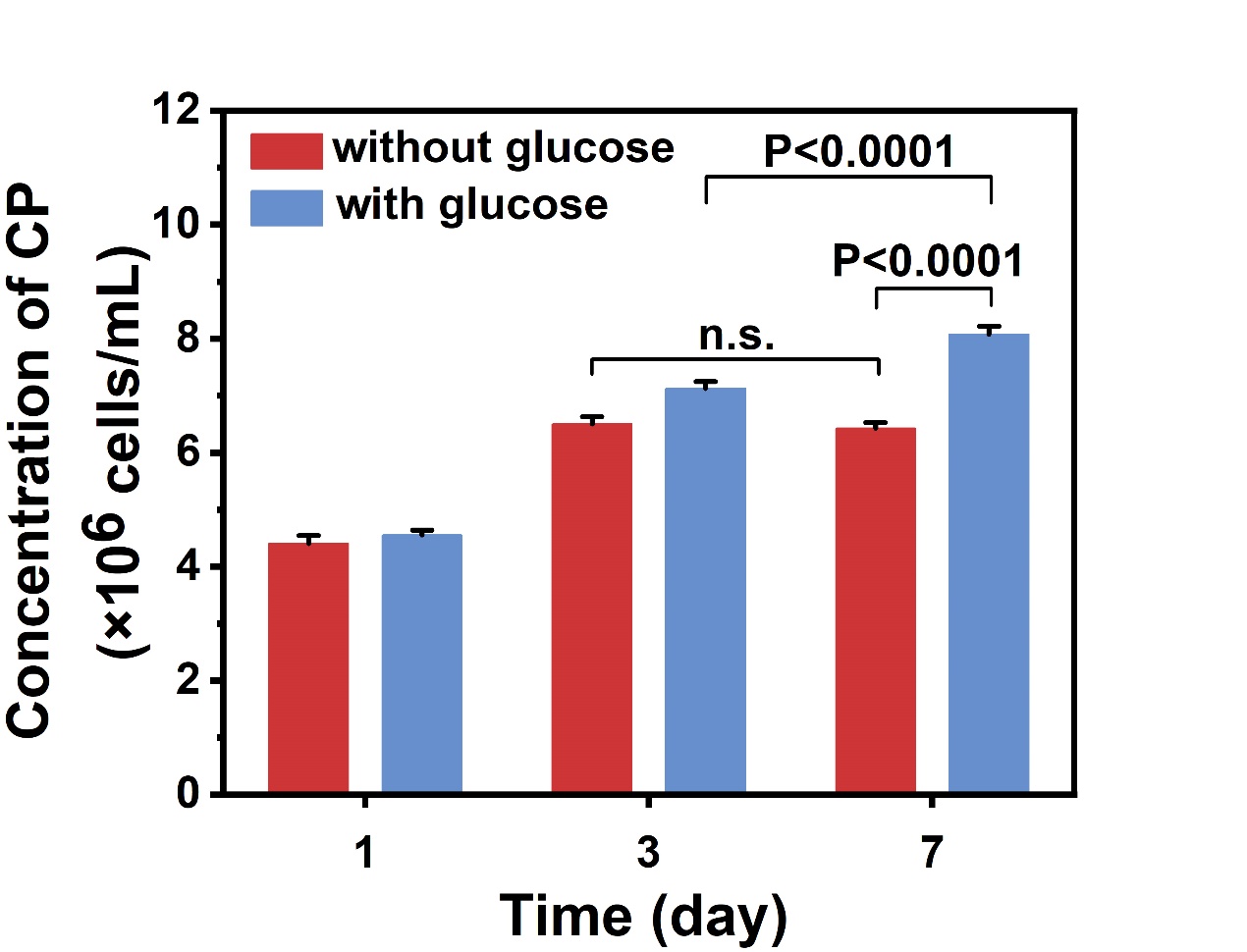


**Figure S2.** The influence of Glucose on the proliferation of CP.

Figure S3


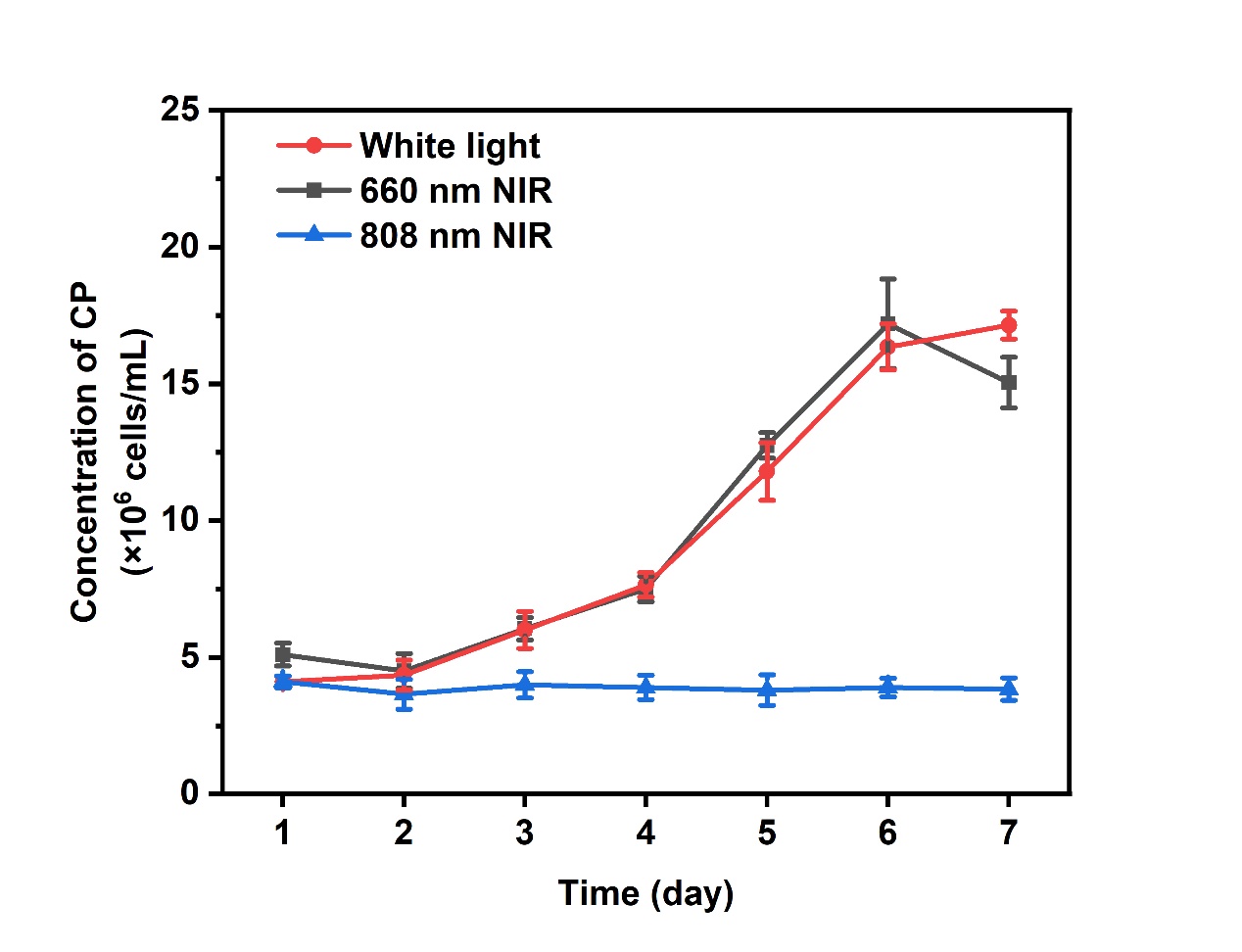


**Figure S3.** Proliferation of CP under different light irradiation.

White light, 808 nm NIR and 660 nm NIR were used to irradiate CP, respectively. The irradiation method was (12 hours on, 12 hours off).

Figure S4


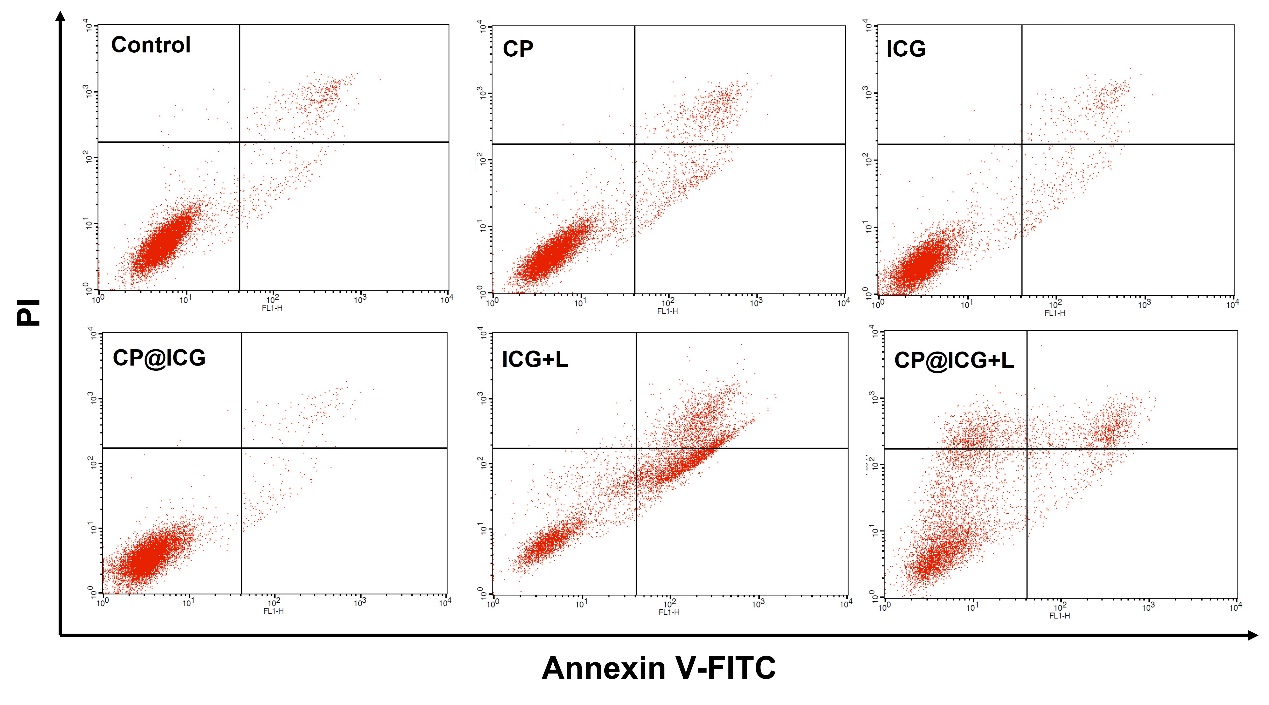


**Figure S4.** Cell apoptosis test under different treatment conditions.
